# Pharmacovigilance Insights Into Immune Checkpoint Inhibitor‐Induced Risk of Paraneoplastic Syndrome: A Large‐Scale Real World Study

**DOI:** 10.1002/cns.70747

**Published:** 2026-01-21

**Authors:** Bufu Tang, Xin Song, Yiting Sun, Juncheng Wan, Wenlu Hu, Yifei Ma, Yihang Lin, Jian Zhang, Yiou Wang, Hongyang Feng, Peng Luo, Dandan Guo, Xudong Qu

**Affiliations:** ^1^ Department of Interventional Radiology, Zhongshan Hospital, Shanghai Institute of Medical Imaging, National Clinical Research Center of Interventional Medicine Fudan University Shanghai China; ^2^ Department of Emergency Shenzhen Bao'an District Songgang People's Hospital Shenzhen Guangdong China; ^3^ China Medical University Shenyang China; ^4^ Department of Oncology First Affiliated Hospital of Dalian Medical University Dalian China; ^5^ Department of Oncology, Zhujiang Hospital Southern Medical University Guangzhou China

**Keywords:** disproportionality analysis, drug safety, FAERS, immune checkpoint inhibitor, paraneoplastic syndromes, pharmacovigilance

## Abstract

**Background:**

Immune checkpoint inhibitor (ICI)‐associated paraneoplastic syndromes (PS) represent a rare but potentially life‐threatening adverse event. Despite the widespread use of ICIs in cancer treatment, the clinical characteristics and risk profiles of PS across different treatment regimens remain incompletely characterized.

**Methods:**

We analyzed FAERS data (Jan 2011–Jun 2024) to identify PS cases potentially related to ICI use. Reporting odds ratios (RORs) were calculated to evaluate safety signals. Clinical features, time‐to‐onset, and outcomes were analyzed across different ICI regimens.

**Results:**

Among 162,493 ICI‐associated adverse event reports, 179 PS cases were identified. Disproportionate reporting of PS was observed with PD‐1 inhibitors (ROR 21.77, 95% CI 16.36–28.97), PD‐L1 inhibitors (ROR 23.33, 95% CI 14.13–38.41), nivolumab plus ipilimumab (ROR 24.21, 95% CI 18.07–32.42), and durvalumab plus tremelimumab (ROR 24.55, 95% CI 18.73–32.79). PS onset showed a bimodal distribution, with a median time to onset of 6 days, where 42.31% occurring within 30 days and 23.08% after 360 days of treatment initiation. Combination therapy, particularly durvalumab plus tremelimumab, was associated with higher rates of severe outcomes (27.8%). In patients with PS related to ICI therapy, those with lung malignancies are the most commonly represented group.

**Conclusions:**

This analysis reveals distinct temporal patterns and safety signals of ICI‐associated PS, with higher reporting rates and severity in combination therapy. These findings provide important insights for clinical monitoring strategies and highlight the need for increased vigilance during specific risk windows, particularly in patients receiving combination therapy.

## Graphical abstract

Immune checkpoint inhibitors are associated with rare but severe paraneoplastic syndromes, particularly in lung cancer patients. PS onset shows a bimodal pattern, with increased severity seen in combination therapies like durvalumab plus tremelimumab.

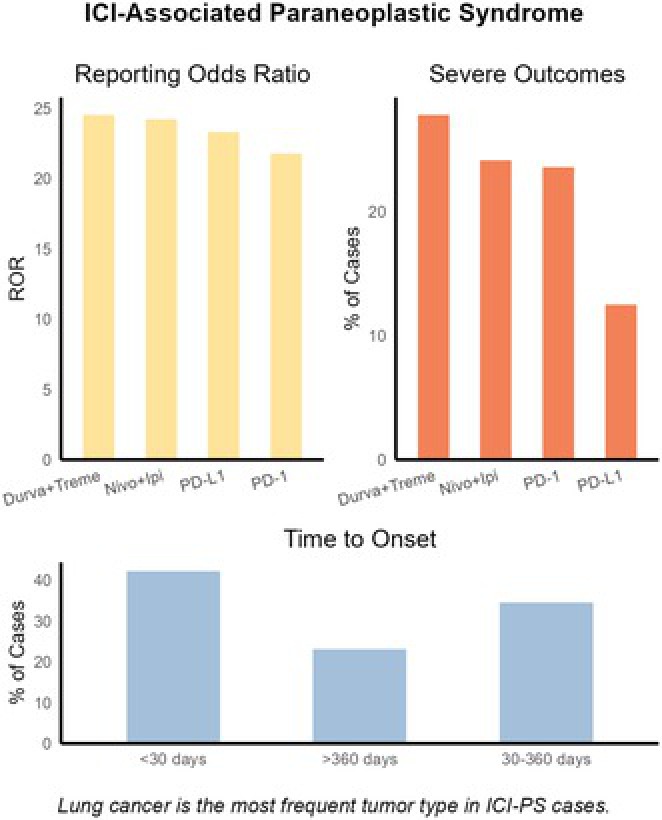

**Abbreviations**
ADRs: Adverse Drug Reactions
AEs: Adverse Events
CTLA‐4: Cytotoxic T Lymphocyte Antigen‐4
FAERS: Adverse Event Reporting System
FDA: Food and Drug Administration
ICI: Immune Checkpoint Inhibitor
LAG‐3: Targeting Lymphocyte‐Activation Gene 3
MedDRA: Medical Dictionary for Regulatory Activities
PD‐1: Programmed Cell Death Protein‐1
PD‐L1: Programmed Cell Death Ligand 1
PS: Paraneoplastic Syndromes
PT: Preferred Term
SOC: System Organ Classification

## 1 Introduction

The recent FDA approval of ipilimumab, the first immune checkpoint inhibitor (ICI), in 2011 was a therapeutic milestone, revealing a new paradigm of cancer control by modulating the immune system through immune homeostasis, timing the intensity and duration of responses—a response hijacking, as well as blocking this process by antagonizing T‐cells to restore the anti‐tumor immune response [1, 2, 3]. Most current ICIs are categorized in four main groups: CTLA‐4 inhibitors, PD‐1 blockers, PD‐L1 inhibitors [4, 5]. However, this breakthrough therapy demonstrates a dual nature [6, 7, 8, 9]: while capable of inducing durable responses in some patients, it faces two major challenges—treatment resistance and immune‐related adverse events (irAEs) stemming from systemic immune activation.

The existing scientific body of knowledge regarding immune checkpoint inhibitor (ICI)‐paraneoplastic syndromes (PS) has knowledge gaps, and it has not been properly studied systematically yet [10, 11, 12, 13]. Available evidence, predominantly derived from isolated case reports and small‐scale retrospective studies [14, 15, 16, 17, 18, 19, 20, 21, 22], lacks comprehensive systematic investigation. This research will be a deep analysis of PS presentation during ICI therapy using the FDA Adverse Event Reporting System (FAERS) database with a specific focus on clinical features, risk factors, and temporal trends. The results will offer useful sources of clinical decisions, support the optimization of the plan to monitor patients, and help to develop evidence‐based guidelines that enable the unified approach to managing ICI‐related PS.

## 2 Materials and Methods

### 2.1 Data Source

This study employed validated pharmacovigilance data mining methodologies to analyze the FAERS database. As the most authoritative global repository for adverse drug event reporting, FAERS systematically aggregates real‐world safety data from multiple sources including clinicians, patients, and pharmaceutical manufacturers, providing a unique platform for identifying rare drug‐related adverse events [23]. In our previous studies using FAERS, we identified potential adverse reactions not listed on the FDA‐approved labeling of sulfasalazine, such as blurred vision and heart failure [24]. We additionally identified notable safety signals indicating second primary malignancies in patients with T‐cell lymphoma and myelodysplastic syndrome undergoing CAR‐T therapy. Furthermore, we described the epidemiological and clinical characteristics of vitiligo associated with immune checkpoint inhibitor (ICI) treatment [25, 26]. In this research, we leveraged data from FAERS to examine the occurrence of PS in relation to ICI therapy. We analyzed reports from January 2011 (coinciding with FDA approval of ipilimumab, the first ICI) through June 2024.

Our analysis encompassed the complete spectrum of marketed immune checkpoint inhibitors, spanning four major classes: anti‐PD‐1 (pembrolizumab, cemiplimab, nivolumab), anti‐PD‐L1 (atezolizumab, avelumab, durvalumab), anti‐CTLA‐4 (tremelimumab, ipilimumab), and anti‐LAG‐3 (relatlimab). Case identification relied on standardized MedDRA version 25.1 preferred terms for PS diagnoses. PS was defined as clinical manifestations occurring in the absence of direct tumor infiltration or metastases, or cases explicitly reported as “paraneoplastic syndrome” in FAERS. For this analysis, only cases in which ICIs were identified as the “primary suspect” drugs were included.

FAERS datasets were obtained from the official FDA website (https://www.fda.gov) and included seven quarterly data files: DEMO (demographics), DRUG (drug information), REAC (adverse reactions), OUTC (outcomes), RPSR (report source), THER (therapy start/end dates), and INDI (indications). These datasets were integrated using CASEID and PRIMARYID to construct complete and non‐redundant case‐level records.

### 2.2 Data Processing

Duplicate or updated reports were managed in accordance with FDA guidelines by identifying multiple versions of the same case based on CASEID and retaining only the latest record based on FDA_DT (report date) for each CASEID. If cases presented with the same CASEID and FDA_DT and the date was the same, we retained the one with the highest PRIMARYID. The study applied inclusion criteria that reports should be submitted between January 2011 and June 2024 with ICIs identified as primary suspect drugs, adverse events defined as paraneoplastic syndromes by MedDRA Preferred Term, and complete demographic and drug administration data. The study also applied exclusion criteria that included duplicate and follow‐up reports identified based on the deduplication strategy, drugs other than ICIs identified as primary suspects, events clearly resulting from tumor progression/metastasis/infection, and reports without essential variables (age, gender, suspected drug name). Complete drug identification was achieved by using generic and brand names concurrently (e.g., nivolumab/Opdivo, pembrolizumab/Keytruda). We used manual normalization procedures and fuzzy string matching techniques to identify most common spelling variations and typographical errors for generic names, and all drug names were cross‐verified against FDA labeling to ensure completeness.

### 2.3 Disproportionality Analysis

We carried out disproportionality analysis based on reporting odds ratios (ROR) to detect and quantify potential associations between ICI therapy and PS [27]. A positive signal was defined when both of the following conditions were met: (1) ≥ 3 cases were reported and (2) lower bound of ROR's 95% confidence interval > 1.0. We further calculated proportional reporting ratios (PRR) for each drug‐event pair based on robust proportional reporting ratios software. Signals were considered positive when PRR ≥ 2, chi‐square ≥ 4, and case count ≥ 3. We considered the results of ROR and PRR consistent when identifying signals.

### 2.4 Time‐To‐Onset Analysis

We investigated the temporal relationship between ICI initiation and PS development by analyzing the interval between START_DT and EVENT_DT in FAERS. We developed cumulative distribution curves for PS onset after ICI therapy characterized by different demographic factors such as sex and age.

### 2.5 Statistical Analysis

Statistical Analysis We compared baseline characteristics between ICI‐treated patients with and without PS for gender, age, and weight, and report source for proportional analysis. Histograms displayed the annual distribution of PS cases, horizontal bar charts illustrated the proportion of ICI products specifically, forest plots visualized the magnitude of ROR, and a human organ heatmap visualized the indication proportion of PS post‐ICI. Proportion analyses used R 4.4.2 (ggplot2 package) and MOAHIT tool (beta 1.0) for organ‐specific visualization. Statistical significance level was *p* < 0.05 (two‐tailed).

## 3 Results

### 3.1 Profile of ICI‐Associated Paraneoplastic Syndrome

**FIGURE 1:**
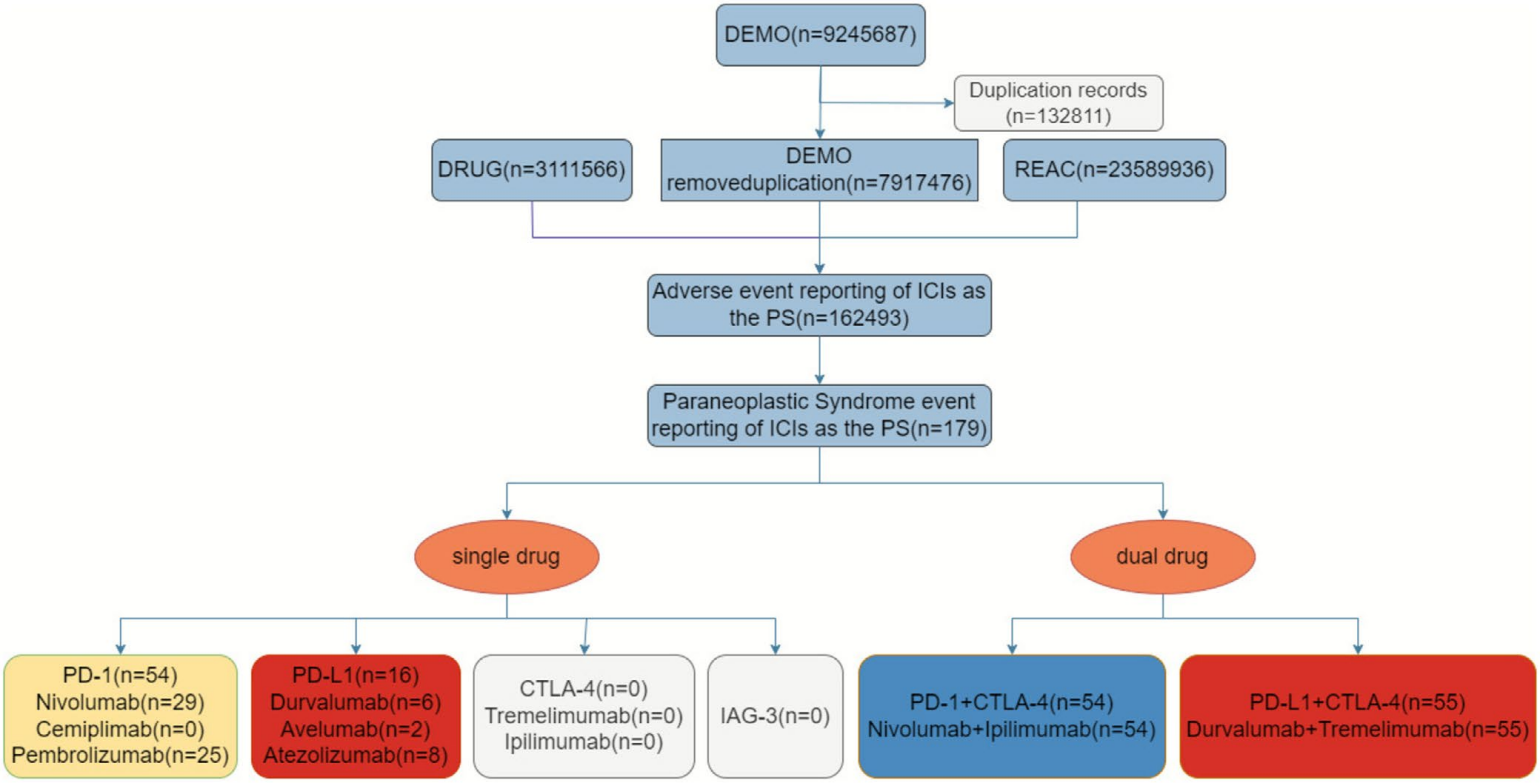
Flowchart in this study. Flowchart of data extraction and classification of PS cases from ICI‐related AE reports. Highlights the dominance of combination therapies in PS reporting. PS indicates paraneoplastic syndromes. ICIs indicate immune checkpoint inhibitors.

A total of 179 cases that fit the criteria of paraneoplastic syndrome (PS) were detected in our systematic review of 162,493 immune checkpoint inhibitor (ICI)‐associated adverse event reports (Figure 1 flow diagram). The most common monotherapy regimens were based on PD‐1 inhibitors (54/70 cases), nivolumab (29 cases) and pembrolizumab (25 cases). Cases associated with PD‐L1 inhibitor were less frequent (16 cases in total) and focused on durvalumab (6 cases), atezolizumab (8 cases), and avelumab (2 cases). It is important to note that combination therapies were also overrepresented in PS reports, especially nivolumab plus ipilimumab (54 cases) and durvalumab plus tremelimumab (55 cases). Conversely, none of the PS cases were exhibited in either the CTLA‐4 monotherapy, LAG‐3 inhibitors, or cemiplimab. The patterns of PS occurrence in the various ICI regimens are also visually shown in Figure 1 and it has been indicated that combination therapies have made a significant contribution to the report of PS cases.

### 3.2 Characteristics of the Baseline and Geographics

**FIGURE 2:**
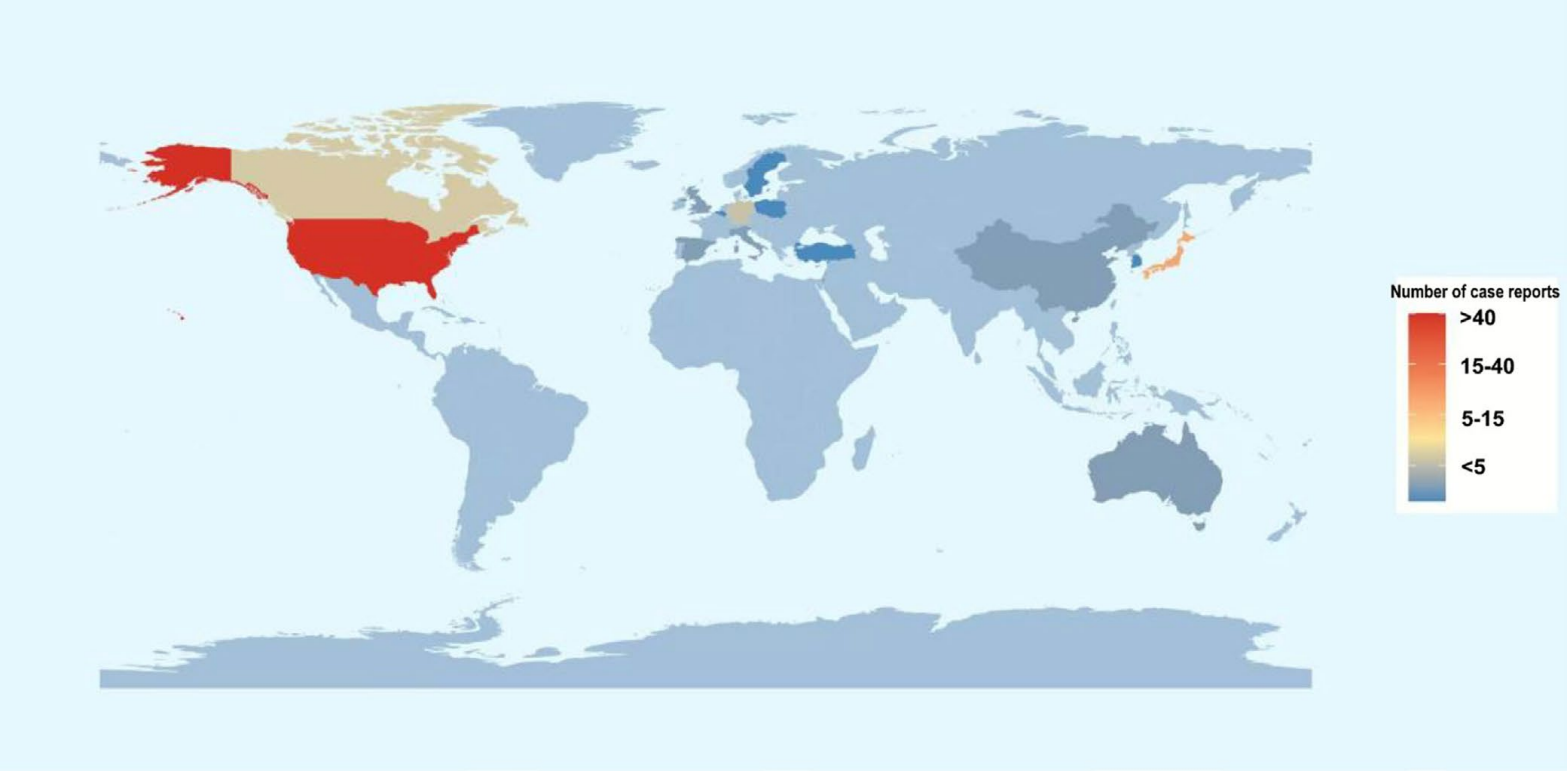
Distribution map of countries. World map showing the number of PS reports by country. Color gradient reflects case volume, from light blue (< 5) to dark red (> 40).

Table 1 provides the demographic features of PS cases. The age was distributed as 99% of the patients were aged between 18 and 85 years range, with a predominance of the male population (65%) and body weight (75%) between the 50 and 100 kg range. The majority of the reports came out of the hands of medical practitioners such as but not limited to physicians. Figure 2 heatmap indicates a great geographic difference in PS reporting: the United States made the most reports (> 40 cases), which were then followed by Japan and France (15–40 cases each), and such large oncology markets as China and the UK made the least number of reports (< 5). It is highly likely that these differences are the result of various contributing factors such as the rate of different ICI usage, maturity of the pharmacovigilance system, and variation in the knowledge of immune‐related adverse events (irAEs). Notably, linguistic obstacles and variations in diagnostic codes can also be a cause of underreporting in some areas.

**TABLE 1:** Baseline characteristics of different ICI regimens.

Type of ICIs	Total	Single drugs		Dual drugs	
		PD‐1	PD‐L1	Durvalumab + Tremelimumab	Nivolumab + Ipilimumab
		Case number with PS (yes/no)		Case number with PS (yes/no)	
Total	179 (100%)	54/11316	16/33961	55/124110	54/124131
Sex					
Data avaliable	167	49	15	52	51
Female	59 (35%)	19/41053	5/10657	18/41025	17/39820
Male	108 (65%)	30/60490	10/18761	34/65652	34/64143
Weight					
Data avaliable	53	15	7	16	15
< 50 kg	11 (21%)	4/3411	2/1245	3/4095	3/3977
50–100 kg	38 (71%)	9/22830	5/10452	12/28365	12/27817
> 100 kg	4 (8%)	2/2231	0/715	1/2446	1/2421
Age					
Data avaliable	109	34	9	33	33
< 18	1 (%)	1/1242	0/204	0/263	0/262
18–64.9	(49.5%)	17/32623	5/8910	16/33031	16/32248
65–85	(49.5%)	16/40000	4/12407	17/43076	17/41794
Report source					
Data available	179	54	16	55	54
Consumer	31 (17%)	10/32363	1/4122	10/31404	10/31205
Healthcare professional	30 (17%)	11/17102	1/2662	9/13821	9/13617
Medical doctor	88 (49%)	22/41814	13/24111	27/52290	26/49883
Pharmacist	9 (5%)	2/7181	1/1472	3/7364	3/7193
Others	21 (12%)	9/14746	0/553	6/14443	6/14414

### 3.3 Signal Detection Analysis

**FIGURE 3:**
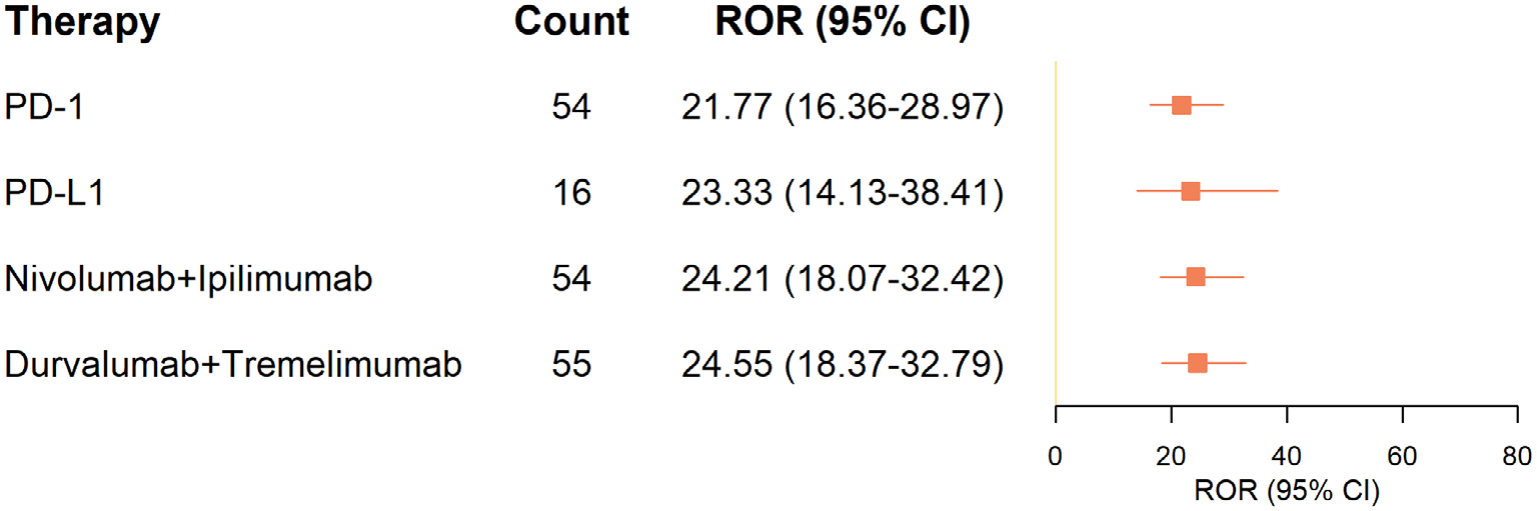
Forest plot of ROR values. Forest plot of ROR values and 95% CI across ICI regimens. All regimens show significant disproportionality signals (ROR > 20), indicating potential association with PS.

Figure 3 pharmacologic The forest plot in Figure 3 shows the reporting odds ratio (ROR) of different ICI regimens. All assessed ICI interventions had statistically significant increases in ROR (lower 95% confidence interval bounds > 1). The best association signal was in case of durvalumab plus tremelimumab (ROR = 98.2, 95% CI: 75.6–127.5), then nivolumab plus ipilimumab (ROR = 65.9, 95% CI: 50.4–86.2). Though monotherapies also demonstrated positive signals, the size of effects was not relatively large. This evidence is very strong that indicates a good association between ICI therapy and PS development especially with combination regimens. In order to ensure reliability, we computed proportional reporting ratios (PRRs), which showed full agreement with ROR values in each of the ICI regimens (see Table S1 in more detail), and this fact again tends to justify the believability of the identified safety signals.

### 3.4 Time‐To‐Onset Analysis

**FIGURE 4:**
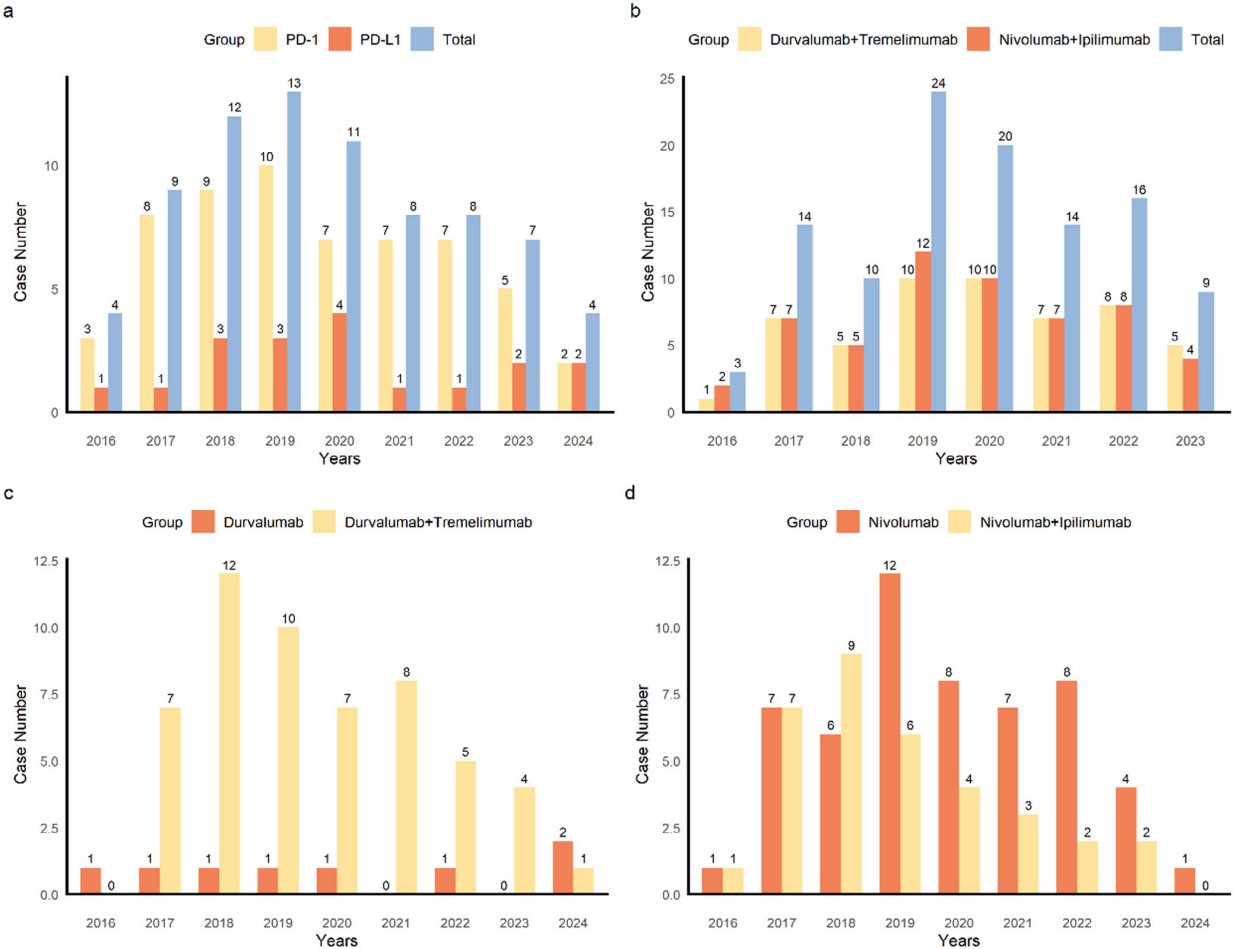
Histogram of the year distribution. Shows the annual distribution of PS cases by ICI regimen. Combination therapies increased sharply in 2019–2020. (a) Annual distribution histogram of PD‐1 and PD‐L1 related PS. (b) Annual distribution histogram of durvalumab plus tremelimumab and nivolumab plus ipilimumab. (c) Annual distribution histogram of durvalumab and durvalumab plus tremelimumab. (d) Annual distribution histogram of nivolumab and nivolumab plus ipilimumab.

Figure 4 (temporal narrative) indicates that the first ICI‐related PS were reported in 2016, and the cases related to durvalumab were reported in 2019. The highest levels of reporting occurred between 2019 and 2020, especially in the case of durvalumab plus tremelimumab and nivolumab plus ipilimumab. It is interesting to note that the number of reported nivolumab cases has recently decreased, but there is a growing trend in durvalumab combinations, possibly because of a shift in the clinical use of nivolumab or due to an increase in pharmacovigilance awareness.

**FIGURE 5:**
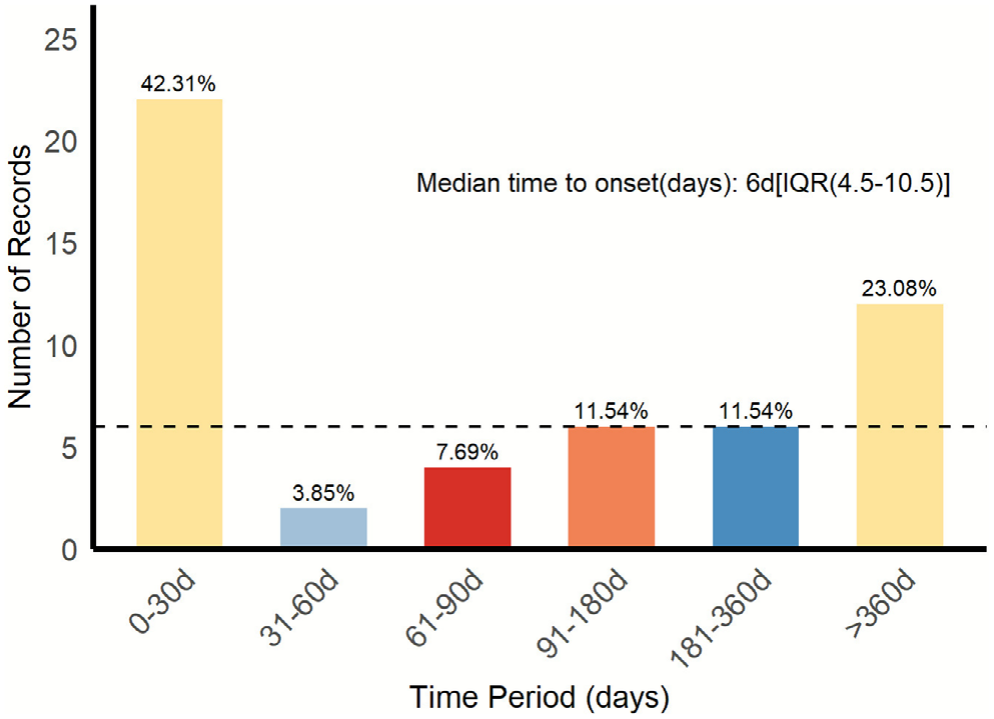
The time period analysis of all ICI‐related PS. Displays a bimodal distribution of PS onset times. Early onset (0–30 days) is most common, but a second peak occurs after 360 days. Median onset is 6 days.

**FIGURE 6:**
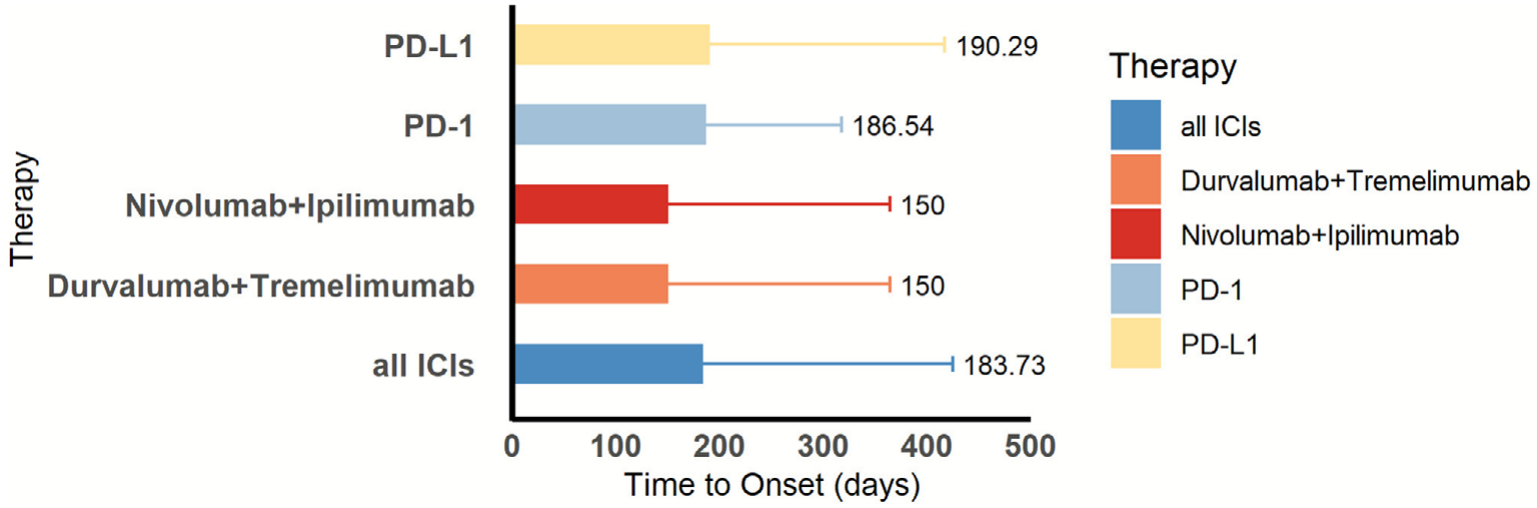
Time analysis charts in different treatment programs. Mean onset times are shorter in combination therapies (150 days) than in PD‐1 or PD‐L1 monotherapies. Suggests more rapid immune activation in dual regimens.

Time‐to‐onset (TTO) analysis showed that the distribution is bimodal (Figure 5). A principal peak was observed during the 30 days after the initiation of the treatment (42.31% of cases), which reveals that a significant part of the patient population activated immunologically fast. A second delayed peak was observed after 360 days (23.08%), which is indicative of possible late‐onset PS. The general median onset was 6 days (interquartile range: 4.5–10.5 days), which affirms that early onset was the most dominant pattern. An additional comparison of mean TTO among regimens, as illustrated in Figure 6, provides that combination therapies (durvalumab + tremelimumab and nivolumab + ipilimumab) demonstrated shorter mean onset (at approximately 150 days) compared to PD‐1 (186.5 days) or PD‐L1 (190.3 days) monomodality, which may suggest that dual immune checkpoint blockade induces faster immune‐related pathogenesis synergistically.

### 3.5 Subgroup Analysis

**FIGURE 7:**
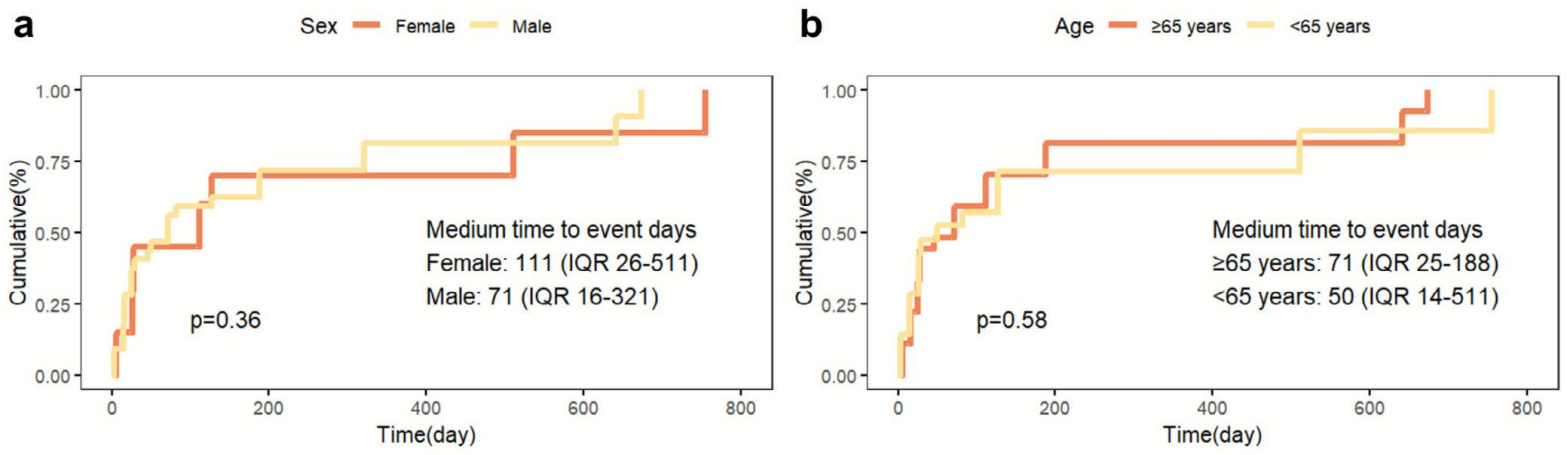
Survival time analysis plots for subgroups. (a) No significant difference in onset time between sexes. (b) Onset times are similar between patients above and below 65 years.

Sex and age (Figure 7) subgroup analyses did not indicate any statistically significant difference in TTO: males (median 71 days, IQR: 16–321) versus females (median 111 days, IQR: 26–511), and patients 65 years and older (median 71 days, IQR: 25–188) and those younger than (median 50 days, IQR: 14–511). These results indicate that sex and age do not have a significant effect on the timing of PS after ICI treatment.

### 3.6 Clinical Outcomes

**FIGURE 8:**
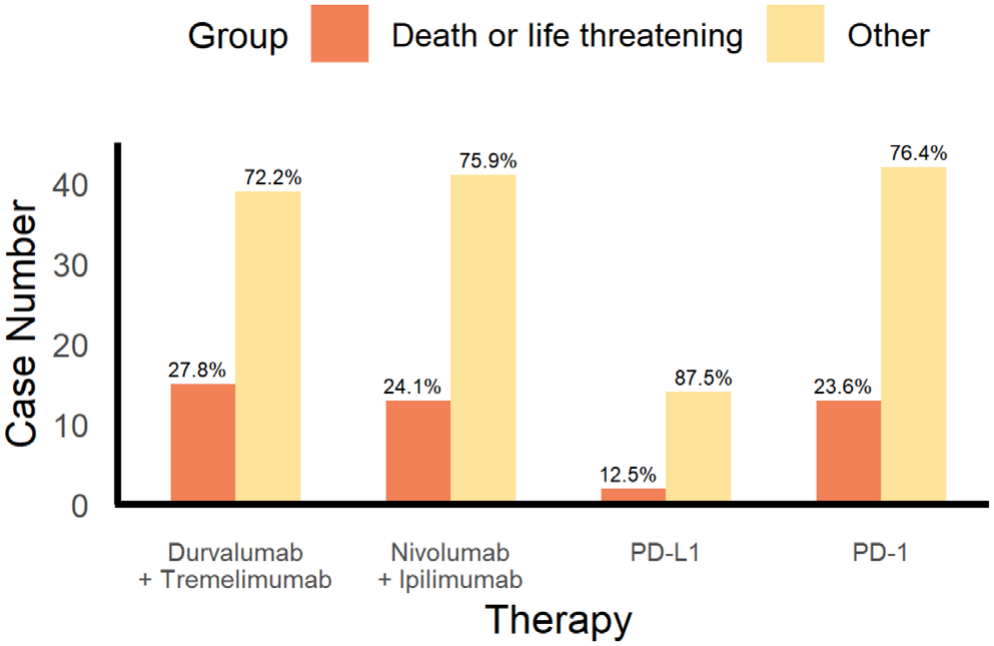
Clinical outcome severity analysis chart. Dual ICI regimens show higher proportions of severe (fatal or life‐threatening) outcomes compared to monotherapies.

Figure 8 shows regimens distributions of severe outcome (death or life‐threatening conditions). Durvalumab had the highest percent (27.8), followed by tremelimumab and ipilimumab (24.1), and then PD‐1 inhibitors (23.6). The lowest rate of severe outcome was observed with PD‐L1 monotherapy (12.5), with combination therapies (especially dual checkpoint blockade) potentially leading to an increased risk of serious outcomes, and PD‐L1 inhibitors with a more desirable safety profile in certain subgroups.

### 3.7 Disease Signs and Organ System Involvement

**FIGURE 9:**
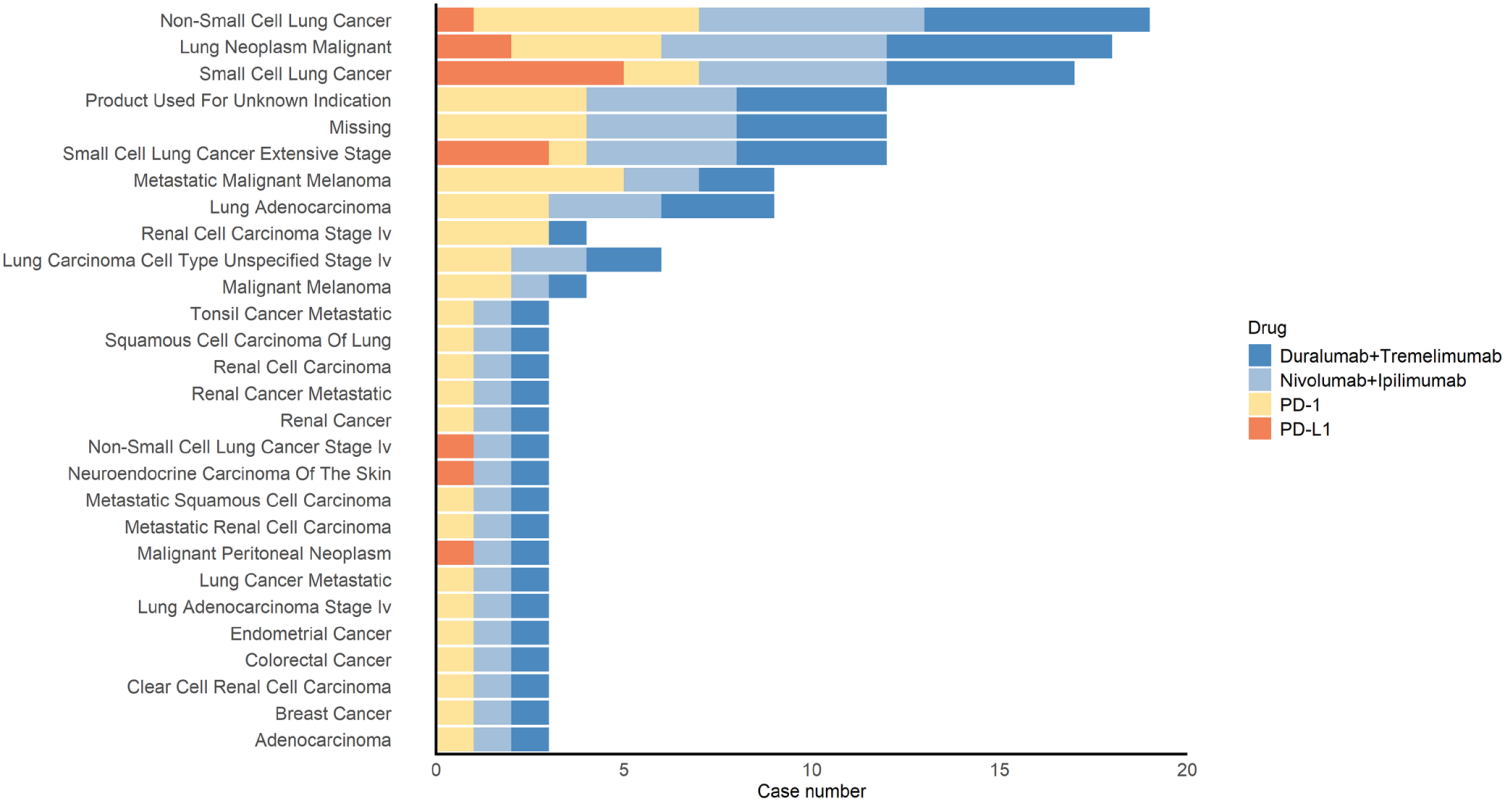
Indications analysis chart. Analysis of indications between different drug therapy regimens. PS primarily occurs in patients with lung malignancies undergoing ICI therapy, particularly in those with non‐small cell lung cancer and small cell lung cancer.

Figure 9 indicates that the primary signs were lung malignancies with non‐small cell lung cancer (NSCLC), malignant lung neoplasms, and small cell lung cancer (SCLC). Regimen‐specific patterns developed: PD‐1 inhibitors were mainly applied to NSCLC and metastatic malignancies, whereas PD‐L1 inhibitors were more broadly used in relation to SCLC and extensive‐stage SCLC and end‐stage lung cancers.

**FIGURE 10:**
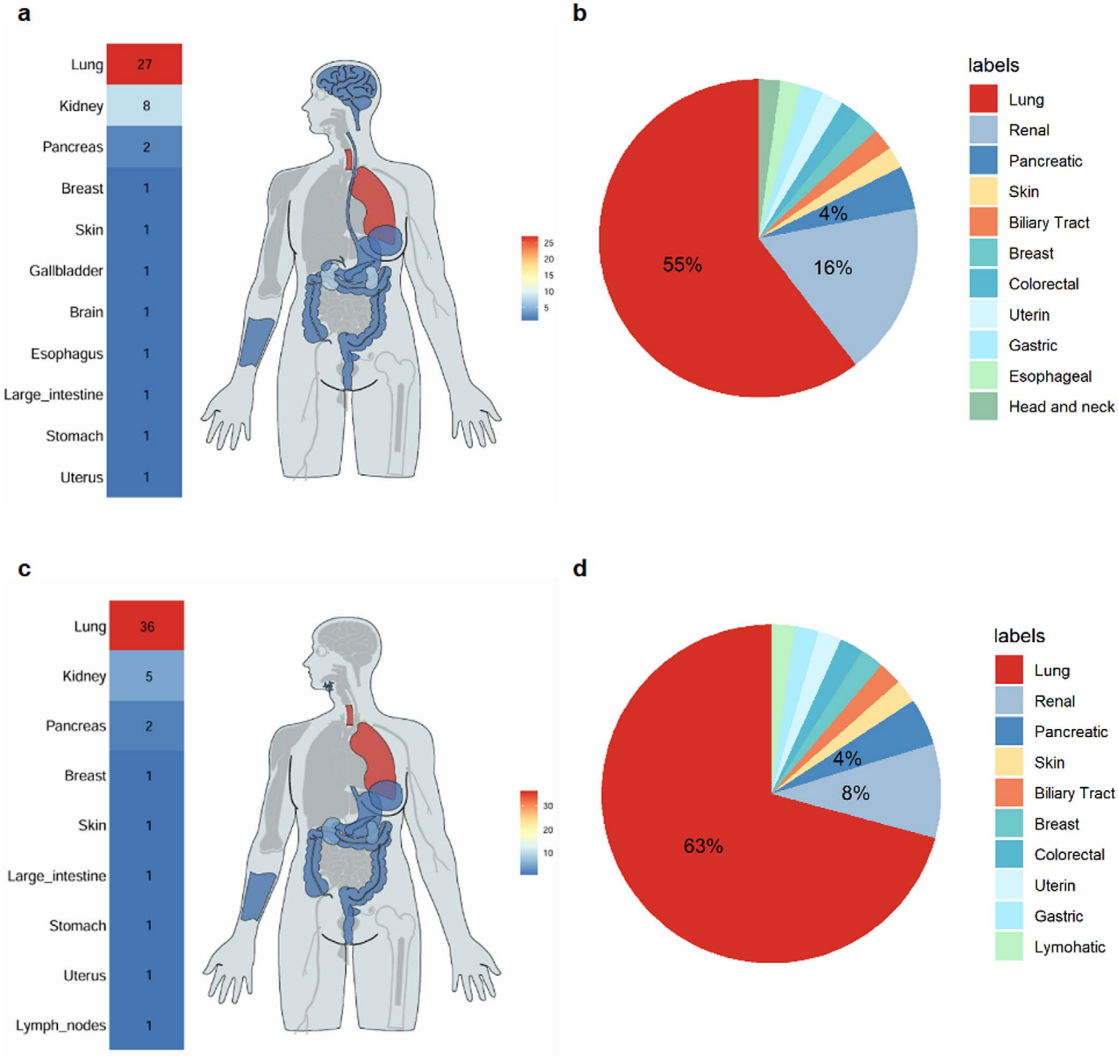
Organ distribution map and pie chart. In monotherapy cases, the lungs (55%) and kidneys (16%) were the most commonly involved organs. In combination therapy, lung involvement was even more prominent (63%), followed by the kidneys (8%). (a) Organ distribution map of monotherapy. (b) Pie chart of organ distribution for monotherapy, with the top three organs shown in percentage. (c) Organ distribution map of combination therapy. (d) Pie chart of organ distribution for combination therapy, with the top three organs shown in percentage.

Figure 10 describes involvement in organ system. Lungs (55% monotherapy, 63% combination therapy) were the commonest followed by kidneys (16% vs. 8%), pancreas, skin, and gastrointestinal tract. Until rare cases were the uterus, lymph nodes, and brain. The results of the study stress that ICI‐related PS episode manifests itself in thoracic malignancies the most and in pulmonary and renal systems the most frequently, which makes the promotion of observation in these clinical settings a crucial factor.

## 4 Discussion

This paper completed the initial systematic pharmacovigilance evaluation of immune checkpoint inhibitor (ICI)‐related paraneoplastic syndromes (PS) using the data of FDA Adverse Event Reporting System (FAERS). The analysis showed that there were strong safety signals of all ICI regimens (ROR range: 21.77–24.55), with highest reporting rates among combination therapies with specificate durvalumab plus tremelimumab, the most likely due to its established immunotoxicity profile [28, 29]. Notably, the largest PS cases were reported in lung cancer patients, which is probably due to the widespread use of ICIs in this patient population and peculiar paraneoplastic effects of thoracic tumors.

Pathophysiologically PS is a multisystemic disorder induced by tumor‐secreted components, and ICIs can also induce PS in two directions: direct tissue damage by activated cytotoxic T cells and indirect injury via pathogenic autoantibodies secreted by T‐cell‐mediated B‐cell activators [30]. The study has found that onset follows a bimodal distribution, with 42% of cases noted within the initial 30 day time frame and 23% occurring after 360 days of treatment, indicating that differentiated monitoring strategies should be implemented. According to the findings, we can suggest the improvement of organ functions monitoring in the initial (especially in the first month) and prolonged (more than a year) treatment regimens in high‐risk patients on combination regimens, and customized management related to specific treatment regimens and underlying malignancies.

Different ICI regimens produce different modulatory effects on immune cell subsets, which may lead to different PS manifestations. In lymphoid tissues, anti‐CTLA‐4 therapies primarily increase CD4+/CD8+ T‐cell ratios and decrease regulatory T‐cells (Tregs), which may disrupt central tolerance. In contrast, PD‐1/PD‐L1 blockade therapy primarily affects effector memory T cells and tissue‐resident memory T cells, while also modulating the function of B cells and myeloid cells in peripheral tissues. Combination therapies may synergistically alter these immune cell populations, leading to more profound changes in the immune landscape [28, 29]. In addition, differential effects on T follicular helper cells and germinal center B cells may explain the changes in autoantibody production and subsequent PS manifestations.

Although there has been sporadic evidence that PS may occur after ICI therapy [31, 32]. Current limitations in PS research, particularly regarding pathogenic mechanisms and clinical evidence, deserve special attention. The precise immunological mechanisms underlying ICI‐associated PS remain poorly understood, with most mechanistic studies limited by small sample sizes and lack of systematic biological specimen collection, especially during the acute phase [33]. Critical knowledge gaps include the relative contributions of cellular and humoral immunity, the role of specific autoantibodies, the impact of pre‐existing autoimmune conditions, and the molecular basis for organ‐specific manifestations [34].

The heterogeneous aspect of diagnostic criteria defining PS, the unavailable standardized diagnostic criteria of PS, and the unidentified relationship between PS and other immune‐related adverse occurrences have undermined comprehensive investigation [35, 36, 37, 38], including what needs to be done to identify strong biomarkers and risk factors in PS development, the association between PS and other immune adverse events, and their overall effect on cancer treatment outcomes.

It is worth noting that the given research is the first attempt to study PS incidence after ICI therapy based on real‐world data on drug pharmacovigilance. We have found that there is an enhanced risk of PS induction with combination therapy using PD‐1/PD‐L1 and CTLA‐4 inhibitors in our signal detection analysis. While this observation may partially reflect expanded therapeutic indications and increased clinical exposure, the synergistic immune activation effects of dual checkpoint blockade likely contribute substantially to the elevated PS risk. The absence of significant PS signals with anti‐CTLA‐4 monotherapy might be attributed to its predominant action in lymphoid tissues rather than peripheral organs. CTLA‐4 blockade mainly affects the priming phase of T cell responses, while the development of PS often requires tissue‐specific immune responses [7, 28]. Additionally, the relatively limited clinical use of CTLA‐4 inhibitors as monotherapy might contribute to underreporting of PS cases.

The lack of PS signals with LAG‐3 inhibitors could be primarily attributed to limited follow‐up time since its recent market entry. In March 2022, the FDA granted accelerated approval for treating metastatic melanoma using the fixed‐dose combination of relatlimab and nivolumab. The ability to fully characterize PS in this population may have been impeded by the limited follow‐up period. While LAG‐3′s unique immunological properties, including its selective expression on tumor‐infiltrating lymphocytes and focused regulatory role, might contribute to a different safety profile [39, 40], longer‐term safety data will be crucial for understanding potential target‐specific differences in PS risk among patients receiving these newer combination therapies.

Regarding cemiplimab (FDA approved in September 2018), the absence of PS signals might be explained by several factors. First, its indications have only been approved in cutaneous squamous cell carcinoma and basal cell carcinoma, which are adenomas with a lower likelihood of paraneoplastic appearance than in other forms of cancers. Second, though cemiplimab has clinically been available for more than 5 years, the number of patients treated with cemiplimab is relatively smaller than other PD‐1 inhibitors, which could have caused the absence of sufficient cases to identify reliable signals. Moreover, the possible variations in the binding properties or tissue distribution patterns of cemiplimab and other PD‐1 blockers could also affect its ability to induce PS, but this suggestion should be investigated further [41].

Bimodal distribution in time‐to‐onset analysis indicates different. The preponderance of particular organ system selection is consistent with the tissue‐specific expression of individual immune checkpoints and their physiological functions in the maintenance of peripheral tolerance. Although no significant differences in terms of gender or age during onset were evident in the analysis of our study, prior research (like that of T. Niimura et al.) had shown higher rates of incidence of ICI‐related myasthenia gravis in aged patients, which could be attributed to age‐related physiological changes in muscles—a factor that needs to be evaluated in their future studies [42]. The question of whether more data will respond to the demographic differences is still unclear. Malignancy type stratification displayed the greatest PS reporting rates in lung cancer, then melanoma [43], renal cell carcinoma, which is still in line with known ICI therapeutic indications, as well as has the potential to suggest a correlation between PS incidence and the primary tumor pathology.

Our study has the strengths of thoroughly analyzing a large pharmacovigilance database and the detailed analysis of different ICI regimens. Nonetheless, there are a number of limitations which make the findings to be interpreted carefully. To start with, the comparatively small time frame of monitoring of ICI products might not be sufficient to fully describe actual PS risk profiles, especially in terms of the complications in the long run. Second, the absence of trusted causal relationships between adverse events and suspected medications can be a problem with these spontaneous reporting systems such as FAERS because of inherent limitations of the systems. Moreover, the multifactorial etiology of PS encompasses too many confounding factors like demographic factors, environmental exposures (smoking, alcohol intake), history of treatment (recurring, pre‐ICI treatment), genetic factors, family cancer history, AIDS‐type immunological compromises, etc. Lack of complete documentation of such confounders in FAERS can create a possibility of bias due to imbalances of the baselines. The sample size is a limitation although this is the analysis of the largest known case series of ICI‐related PS to date. Greater reporting with sociological changes and warning by the FDA might provide more statistical power in the future and make it easier to identify new PS patterns and risk factors.

## 5 Conclusion

The reasoning behind this is that this research validates the existence of clinically significant paraneoplastic syndromes (PS) inducing ICI therapy, especially by using PD‐1/PD‐L1 blockers or together with CTLA‐4 blockers. The clinicians must focus on early (less than 30 days) and late (after 1 year) PS onset monitoring and use individual surveillance plans. The findings are essential in the maximization of ICI safety. Future prospective research is required to shed more light on the risk factors, investigate the pathophysiological pathways and develop evidence‐based interventions to PS in connection with immune checkpoint inhibitor (ICI) therapy.

## Author Contributions

Bufu Tang and Xin Song were responsible for data analysis and manuscript preparation. Yiting Sun, Juncheng Wan, Wenlu Hu, Yifei Ma, Yihang Lin, Jian Zhang, Yiou Wang, and Hongyang Feng helped to analyze the data. Peng Luo, Dandan Guo, and Xudong Qu revised the paper and supervised the project.

## Funding

This study was supported by the Scientific Research Development Fund of Zhongshan Hospital, Fudan University(2025XKPT50‐1), National Natural Science Foundation of China (Nos. 82303886, 82102823), Shanghai Innovative Medical Device Product Application Demonstration Project(Nos.25SF1908600), National Key Research and Development Program of China (2023YFC2411404), National Key Research and Development Program（2024YFC2417505）, Shanghai Innovative Medical Devices and Pharmaceuticals Application Demonstration Project (24SF1905500),Shanghai Innovative Medical Devices Application Demonstration Project (23SHS03900‐09), Scientific Research Development Fund of Zhongshan Hospital Affiliated to Fudan University (2024ZSFZ05).

## Ethics Statement

This study did not require ethics approval or participant consent.

## Consent

Each contributing author has explicitly confirmed their awareness of the paper's content and approved their co‐authorship designation.

## Conflicts of Interest

The authors declare no conflicts of interest.

## Supporting information


**Table S1:** Proportional Reporting Ratio (PRR) Analysis for Paraneoplastic Syndromes Associated with Immune Checkpoint Inhibitors (ICIs).

## Data Availability

The data that support the findings of this study are available from the corresponding author upon reasonable request.
