# Supplementary material for: Pharmacovigilance Insights Into Immune Checkpoint Inhibitor‐Induced Risk of Paraneoplastic Syndrome: A Large‐Scale Real World Study
Source: CNS Neurosci Ther. 2026 Jan 21;32(1):e70747. doi: 10.1002/cns.70747 (PMC12820717; doi:10.1002/cns.70747)
Supplement: Supplementary file 1 — Table S1: Proportional Reporting Ratio (PRR) Analysis for Paraneoplastic Syndromes Associated with Immune Checkpoint Inhibitors (ICIs). [file CNS-32-e70747-s001.docx]

**Supplementary Table S1.**

***Proportional Reporting Ratio (PRR) Analysis for Paraneoplastic Syndromes Associated with Immune Checkpoint Inhibitors (ICIs)***

| **ICI Regimen** | **Number of PS Cases** | **PRR** | **Chi-square** | **Signal Detected** |
| --- | --- | --- | --- | --- |
| PD-1 | 96 | 21.76 | 931.84 | Yes |
| PD-L1 | 41 | 23.29 | 328.32 | Yes |
| Nivolumab + Ipilimumab | 84 | 24.2 | 1000.34 | Yes |
| Durvalumab+Tremelimumab | 66 | 24.54 | 1033.8 | Yes |
